# Role of growth rate on the orientational alignment of *Escherichia coli* in a slit

**DOI:** 10.1098/rsos.170463

**Published:** 2017-06-21

**Authors:** Julian Sheats, Bianca Sclavi, Marco Cosentino Lagomarsino, Pietro Cicuta, Kevin D. Dorfman

**Affiliations:** 1 Department of Chemical Engineering and Materials Science, University of Minnesota—Twin Cities, 421 Washington Avenue SE, Minneapolis, MN 55455, USA; 2 LBPA, UMR 8113 du CNRS, École Normale Supérieure de Cachan, Cachan, France; 3 Sorbonne Universités, Université Pierre et Marie Curie, 4 Place Jussieu, Paris, France; 4 CNRS, UMR7238 Computational and Quantitative Biology, Paris, France; 5 IFOM Institute for Molecular Oncology, Milan, Italy; 6 Cavendish Laboratory, University of Cambridge, Cambridge CB3 0HE, UK

**Keywords:** microchemostat, bacteria colony, nematic alignment, microfluidics

## Abstract

We present experimental data on the nematic alignment of *Escherichia coli* bacteria confined in a slit, with an emphasis on the effect of growth rate and corresponding changes in cell aspect ratio. Global alignment with the channel walls arises from the combination of local nematic ordering of nearby cells, induced by cell division and the elongated shape of the cells, and the preferential orientation of cells proximate to the side walls of the slit. Decreasing the growth rate leads to a decrease in alignment with the walls, which is attributed primarily to effects of changing cell aspect ratio rather than changes in the variance in cell area. Decreasing confinement also reduces the degree of alignment by a similar amount as a decrease in the growth rate, but the distribution of the degree of alignment differs. The onset of alignment with the channel walls is coincident with the slits reaching their steady-state occupancy and connected to the re-orientation of locally aligned regions with respect to the walls during density fluctuations.

## 1. Introduction

Bacteria are rod-like organisms, and their spatial orientation provides an ideal opportunity to connect topics in statistical physics to microbiology. When a suspension of rods reaches a critical density, it undergoes an isotropic to nematic phase transition [1,2], a phenomenon that finds widespread application in the physics of liquid crystals [3]. The phase behaviour changes markedly when such systems are confined [4,5]. Bacteria also exhibit nematic alignment, both when growing in a colony [6–8] (due to the density of the colony) and under spatial confinement [9–11]. Organization within bacterial colonies is an area of broad interest in the study of bacterial growth and adaptation. Examples include the role of direct cell to cell contacts in horizontal gene transfer [12] or contact-dependent growth inhibition (CDI) [13], and the control of cell physiology and biofilm development by the diffusible signals of quorum sensing [14].

The present contribution focuses on understanding how the spatial confinement of a microchemostat couples with the growth rate to affect the global alignment of bacterial microcolonies with respect to the channel walls. Microchemostatic devices [9,11,15–31] permit the study of bacteria growth and dynamics at the single-cell level under balanced growth conditions by continuously feeding nutrients, removing waste and maintaining the cell population at an approximately constant number to sustain a particular growth phase, most often exponential growth. Microchemostats generally involve some type of confinement, providing an ideal opportunity to study ordering of bacteria in confinement under fixed growth conditions.

There have been a number of studies looking at microcolony growth and organization, including cell orientation, with an emphasis on the physical perspective [6,8,32–36], including work specifically devoted to studying ordering in a microchemostat [9,11,19]. In particular, early work studying the growth of bacteria in monolayers with microfluidic chemostats [9] found that the layers are ordered, even many cell diameters away from the walls, and that the ordering arises even in relatively complicated chamber shapes. Analysis of the glucose concentrations suggests that ordering removes diffusive transport limitations [9]. Accompanying simulations of this system suggests that the ordering arises solely due to the combination of cell growth and mechanical interactions between cells and between cells and the walls, in the absence of any chemical signalling factors [9]. A separate study, using a nematodynamics model of liquid crystals [37] adapted to account for cell growth, suggested instead that the ordering arises due to the exit flow created by the exponentially increasing mass of bacteria within a slit [11].

**Figure 1.**
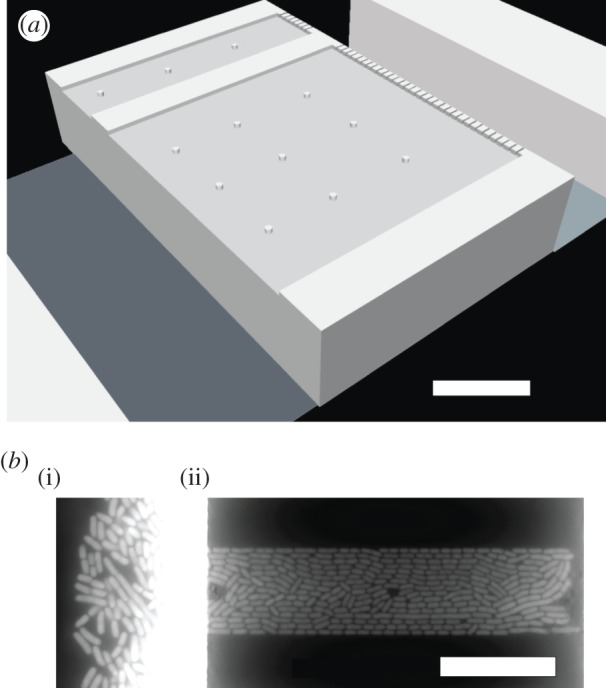
Slit microchemostat and alignment of *E. coli* therein with respect to the channel walls. (*a*) Schematic of two different width slits in the array. The bacteria grow inside the slits, while the adjacent flow channels provide growth media and remove waste and extra cells. The filter at the right side of the slit aids in loading the cells by differential pressure between the two flow channels. Small pillars are included to support the ceiling of the device. The figure is approximately to scale. (*b*) Typical fluorescent image showing one media channel (i) and cells in a narrow slit (ii) grown under fast growth rate (LB). The bright region corresponds to overgrowth in the media channels; a similar overgrowth occurs in the media channel on the right (not shown). White scale bars are 20 μm in both panels.

In what follows, we build on the previous work on nematic alignment in microchemostats [9,11] to understand the interplay between spatial confinement and growth rate. In particular, we know that changing growth rate changes the cell aspect ratio [27] by changing both the average length and average width of a cell [38–40], and simulations [9] indicate that reducing the aspect ratio reduces the orientation of confined bacteria colonies. We have designed the microchemostat depicted in figure 1*a*, which allows us to easily study these factors at the single-cell level. The slits within the device are relatively short, and numerical solutions of the nematodynamic equations in a related system suggest will lead to laminar flow therein [11]. Our device, which was originally designed as an improvement over our previous devices [26,27] for studying gene expression, differs from those used previously to study ordering of confined bacteria. The geometry is simpler than the confinement used by Cho *et al.* [9], permitting a simple analysis of the degree of orientation. It also differs from the devices used by Volfson *et al.* [11] and subsequent work by this group [19,21], which had either one or two open ends. Our use of a filter on one end makes loading the device very simple, since the bacteria can be pushed against the filter by a pressure difference between the two flow channels, while also allowing the bacteria to escape (albeit not easily) from the nominally ‘closed’ end of the device.

## 2. Experimental methods

### 2.1. Bacterial culture

The *E. coli* strain in these experiments contains the *mut2gfp* gene under control of the *rrnB*P1 promoter [27]. Cultures were grown overnight from streaked agar pads resting at 4°C in either Luria Broth (LB) or M9 minimal liquid media supplemented with 0.2% casamino acids at 37°C along with 0.1% kanamycin. These were then diluted 200× in liquid media and allowed to grow to an OD_600_ of at least 0.1 at 37°C. Roughly 50 μl of this suspension was then added to tubing attached to the microchemostat. Care was taken to reduce thermal shock to the cells by keeping the device near 37°C.

### 2.2. Microchemostat fabrication

The silicon and photoresist two-layer master was created using standard photolithographic methods. The first layer was etched into the wafer, and the second was created bottom-up using a 19 μm thick layer of AZ 9260 photoresist. The first layer involved 1.0–1.2 μm deep slits of varied width bounded by a filter at one end, which has approximately 0.5 μm wide holes to prevent cells from escaping the slits on that end. On both sides of these slits, the second layer formed the 19 μm deep, 50 μm wide media channels. These are shown in figure 1*a*. PDMS replicas (Sylgard 184) were cast from this master. The individual devices were cut to size, and inlet and waste holes were punched using sharp hole punches to prevent PDMS debris from clogging the device. The PDMS device was cleaned with ethanol. The glass coverslip (Fisher) was cleaned with a Bunsen burner to remove the commercial coating. Both the device and coverslip were finally cleaned in an air plasma (Harrick) for 30 s. Immediately following this step, in order to preserve the silanol groups on the PDMS and glass surfaces, an individual device was bonded on a hotplate at 105°C for 5 min, tubing was attached to the ports and a 5% solution of poly(ethylene glycol)-terminated silane (Gelest) in ethanol was filled into the channels and slits as the first step of surface passivation. This was allowed to soak for 2 h, after which unbound silane was rinsed out for 1 h using ethanol at $5\;\text{μ}l\;\min^{-1}$. Following this, a 20 mg ml^−1^ bovine serum albumin (BSA) in water solution was filled into the device and allowed to soak for 1 h at 37°C. This solution was then rinsed out using the running growth media containing 5% BSA. This two-step passivation together with a fast flow rate of at least $2.5\;\text{μ}l\;\min^{-1}$ during growth in the device were necessary to prevent cell adhesion to the device and overgrowth biofilm formation.

### 2.3. Imaging

Cells were imaged using the same procedure and set-up we have used previously [27] with the following changes. Autofocus was obtained using an ASI CRISP system (Applied Scientific Instrumentation). This system was calibrated for each device at the start of each experiment. A Leica 63× oil immersion objective was used to increase the intensity of the CRISP infrared beam reflection off of the water/glass interface of the device relative to the 100× objective we used previously. The exposure duration of the fluorescence images was 500 ms.

A passivated device was affixed to an aluminium chuck with aluminium tape. A thermocouple was inserted into a previously punched hole near the media channels, and an additional thermocouple was attached to the aluminium chuck. These were attached to a temperature controller which was joined to a heat source wrapped around the objective. Cells were loaded into the media channel on the open end of the slits and forced into the slits using a higher flow rate by hand. A syringe pump was then used to control the media flow, with a flow of $3.5\;\text{μ}l\;\min^{-1}$ in the media channel on the open end of the slits and $2.5\;\text{μ}l\;\min^{-1}$ in the media channel at the filter end of the slits.

### 2.4. Data analysis

From the overnight time-lapse imaging, a stack of images was obtained for analysis. For each slit, each image chosen was at least one mean cell division time later than the previous and following images; the duration between images of individual slits varied due to poor focus in some images, which were not included for analysis [27]. Images were cropped to contain individual slits. These image portions were then fed into the open-source CellProfiler software to perform cell segmentation and then measurements of orientation.

Orientation was found to be significantly affected by the presence of filamentous cells. As a result, images in the stack were filtered for the presence of a long filament. This was done in an automated way by determining the size of all cells in an image and then removing any image that contained a cell whose long axis was longer than four times the median of all cells in the population at each growth condition. This removed roughly 20% of the images for the narrow slits and 70% of the images for the wide slits. Fortunately, microchemostats have high data throughput, so we still obtained large datasets for ensemble averaging.

Ordering in a two-dimensional nematic is described by the tensor [41]

2.1 $$\text{Q}=\left[\begin{array}{cc} \left\langle \cos2\theta \right\rangle & \left\langle \sin2\theta \right\rangle \\ \left\langle \sin2\theta \right\rangle & -\langle \cos2\theta \rangle \end{array}\right],$$

where *θ* is the angle between the long-axis of the cell and the slit wall. The resulting order parameter is given by

2.2 $$\phi =\sqrt{\frac{1}{2}\mathrm{Tr}({\text{Q}}^{2})}={\left[{\left\langle \cos2\theta \right\rangle }^{2}+{\left\langle \sin2\theta \right\rangle }^{2}\right]}^{1/2}.$$

For a perfectly ordered system, *θ*=0 and *ϕ*=1. Conversely, *ϕ*=0 for a disordered system with *θ* uniformly distributed on [0,*π*]. In what follows, we will take each image as a measurement of the order parameter *ϕ* and thus compute the average 〈⋯ 〉 over the cells within that image.

Note that nematic ordering sometimes involves an aligning field. However, there is evidence for orientation of macroscopic systems in the absence of such a field that are described by equilibrium statistical mechanics [42]. In our case, the force exerted by the walls breaks the symmetry, which propagates inward to create an apparent nematic order, analogous to what happens for a tightly confined semiflexible chain and a tube [43].

## 3. Results and discussion

The operation of the microchemostat in figure 1*a* involves first loading a handful of cells against the filter. These cells then begin dividing, eventually filling the slit. The onset of alignment with the walls is coincident with the onset of the typical cell occupancy at steady-state, consistent with previous work [9] that reported the initially disordered system becoming ordered due to packing of an open chamber.

**Figure 2.**
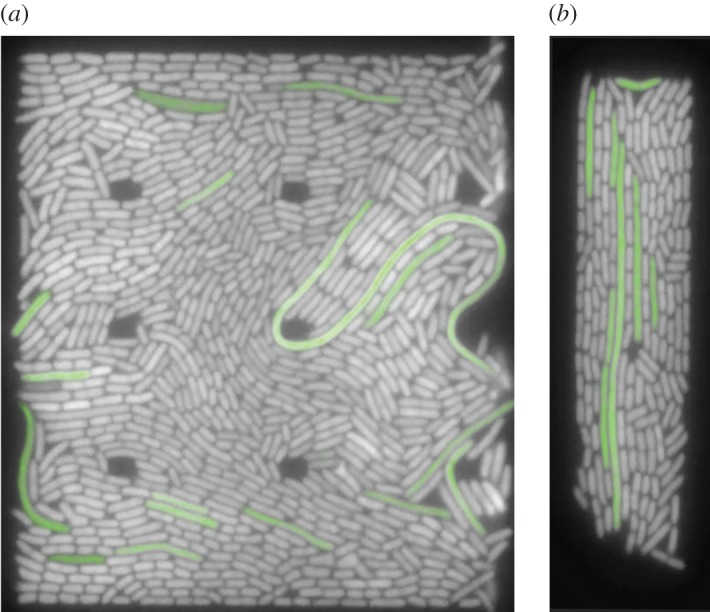
Example images of filaments affecting ordering of cells. Filamentous cells have been false-coloured green. (*a*) Fast/wide and (*b*) fast/narrow.

We focus here on how the steady-state alignment of the bacteria with the channel walls changes as we either increase the slit size or decrease the growth rate. As mentioned in the Experimental methods, filamentous cells in many cases disrupted order (figure 2*a*), while in some cases they appeared to either act neutrally or possibly improve ordering (figure 2*b*). This occurred until the filament left the slit, after which the order returns to the average steady-state value. The potential for anomalously low ordering in the presence of filaments is the reason such images were removed from the analysis.

**Figure 3.**
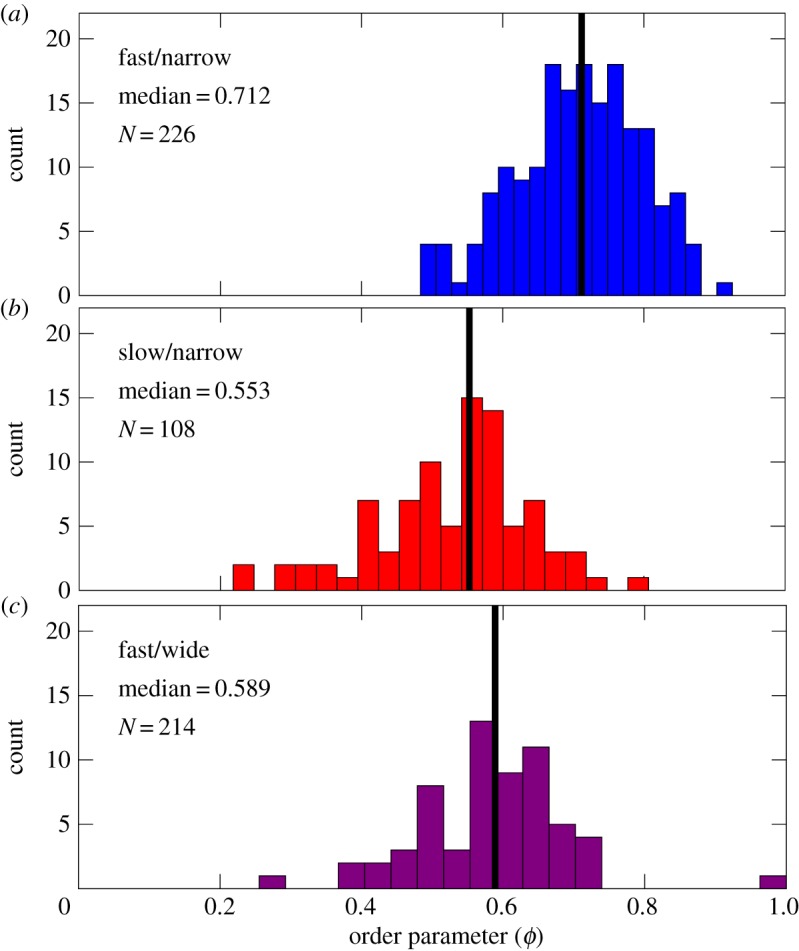
Steady-state alignment of bacteria with the slit walls decreases when either decreasing the growth rate or increasing the slit width. The histograms show the distributions of the order parameter *ϕ* after the slit has been filled and frames containing filamentous cells have been removed. The data for slow/narrow and fast/wide correspond to measurements made over multiple slits and multiple time points within the same experiment; the fast/narrow data correspond to measurements obtained over many slits in two separate experiments. The panels correspond to (*a*) fast growth rate, LB, in narrow slits (15 μm wide), (*b*) slow growth rate, M9+0.2%CAA, in narrow slits and (*c*) fast growth rate in wide slits (60 μm wide). Black vertical lines indicate the median of the distribution.

Figure 3 shows the distribution of the order parameter *ϕ* obtained over three different sets of conditions after removing frames with filamentous cells. While most of the cells are oriented parallel to the walls, we observed cells exhibiting the full range of angles *θ* in our system. Comparing figures 3*a* and 3*b* shows that reducing the growth rate noticeably reduces the degree of ordering. The median value of the distribution at lower growth rate is only 75% that for the faster growth rate in the narrow channels. Increasing the slit width from 15 μm wide (figure 3*a*) to 60 μm wide (figure 3*c*) has a similar effect on the median of the distribution as does changing the growth rate. The persistence of nematic alignment even when the slit is very wide is consistent with previous observations [9].

The decrease in nematic alignment upon increasing the width of the slit is expected. Analogous to the behaviour of worm-like chains in slit confinement [44], the nematic alignment proceeds from a boundary layer near the slit walls and propagates inward. As the slit becomes larger, the effect of the boundary layer becomes increasingly less important, leading to a reduction in the overall alignment.

**Figure 4.**
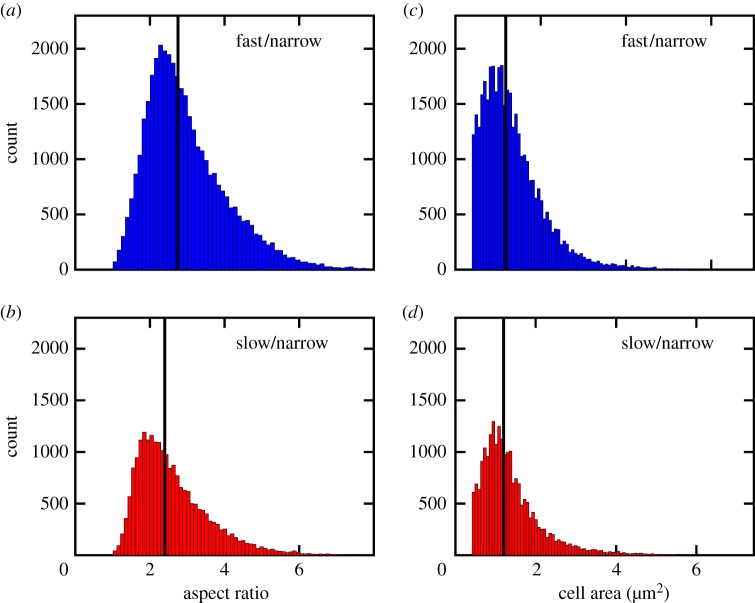
Reducing the growth rate reduces the cell aspect ratio and narrows the distribution in cell area. The figures show distributions of aspect ratio (*a*,*b*) and cell size (*c*,*d*) in narrow slits (15 μm wide) for fast growth (LB, (*a*,*c*)) and slow growth (M9+0.2%CAA, (*b*,*d*)). The black lines indicate the medians in the distributions: aspect ratio—fast/narrow: 2.8; slow/narrow: 2.4. Cell area—fast/narrow: 1.3 μm^2^; slow/narrow: 1.2 μm^2^.

The influence of the growth rate on nematic alignment is less obvious, as the growth rate affects both the cell aspect ratio and the cell size [39,40]. To understand which effect is more important for controlling alignment, we plot the distribution of both quantities in figure 4. Reducing the growth rate reduces the average cell aspect ratio. This effect should lead to a reduction in order for a system of rods, as the isotropic–nematic transition is favoured by the anisotropy of the object [2], and it is consistent with simulations of purely biomechanical interactions in a system of growing rods [9]. The effect of growth rate on cell size is less dramatic, with a slight decrease in the mean area. This effect should favour higher alignment at slower growth rates, as the smaller cell size implies more cells per unit width (thereby reducing the effect of the orientation boundary layer).

On the basis of the data in figures 3 and 4, we can conclude that the reduction in the cell aspect ratio is the dominant reason for the reduction in alignment with the walls at slower growth rates. This conclusion extends previous simulation work [9], which considered changes in aspect ratio that arise by increasing the cell length at a fixed cell diameter.

**Figure 5.**
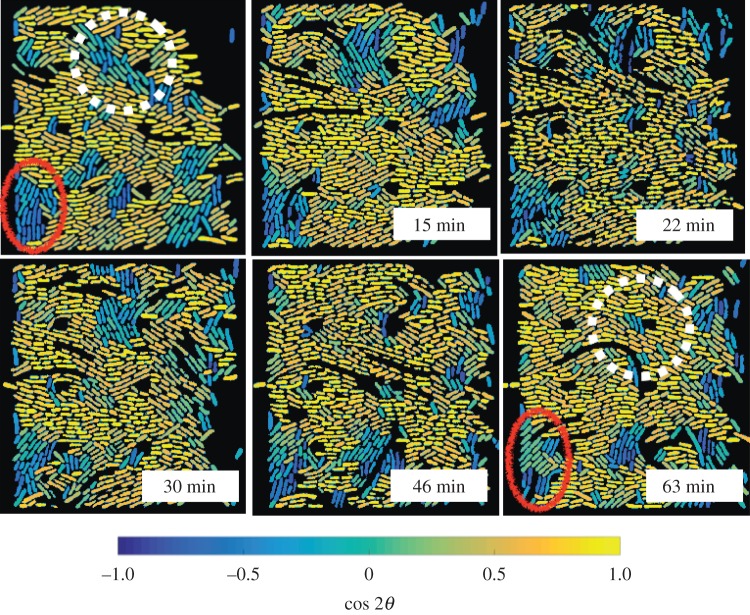
The evolution of domains of low nematic ordering are heterogeneous. The images are snapshots where the segmented cells have been coloured according to the value of cos⁡2θ with respect to the channel walls. The times correspond to the elapsed time since the upper-left frame. The data correspond to fast growth (LB) with a mean doubling time of 18 min in a wide slit (60 μm). Two regions of low ordering with respect to the walls (but strong local nematic ordering) are indicated by the solid red oval and dashed white line. The corresponding movie data are provided as electronic supplementary material.

It is also worthwhile to examine how the ordering arises within a slit. Figure 5 shows a representative result for fast growth in a wide slit, the system which exhibits the most varied behaviour among our three cases. The upper-left panel corresponds to an arbitrary time *t*=0 after the slit has reached its steady-state occupancy. Two different regions of low alignment with respect to the channel walls are indicated in the figure, one localized near the lower-left corner of the slit and another within the bulk of the slit. As the system evolves, the cell density within the latter domain undergoes a strong fluctuation towards lower density. This fluctuation opens up free space, allowing the cells to reconfigure. As these cells are located proximate to cells near the wall, they reorient their long axes to be parallel with the wall. This reorientation is not an instantaneous (nor complete) effect. Nevertheless, the end result is the loss of the domain with poor alignment with respect to the walls, with only a few cells that persist unaligned with the walls.

These dynamics contrast with the region in the lower-left corner of figure 5, where the low alignment with respect to the walls persists throughout the hour-long time period. We propose that this persistent low alignment arises for two reasons. First, there is no density fluctuation that permits the cells to rearrange. Overall, we would expect fewer density fluctuations within the corner of a slit since the cells cannot grow through the walls. Second, and perhaps equally important, the corners of the slit have a competition between alignment parallel to the lower wall of the slit and alignment parallel to the filter at the backside of the slit. The persistence of low alignment in the quasi-closed end of our device is also consistent with theory of Boyer *et al.* [19] that a buckling instability at the closed end of a device inhibits complete nematic alignment. Our system is somewhat more complicated, as both fluid and bacteria can escape through the slit, albeit not as easily as they escape through the open end.

Figure 5 highlights the domain dynamics within a given example. Given the heterogeneity within this particular example, it is reasonable to assume that similar complex dynamics occur throughout the system.

## 4. Concluding remarks

We have examined the effect of growth rate on alignment of bacteria in a slit channel, building on previous work [9,11,19] on the role of confinement on ordering of bacteria. We found that the reduction in alignment with respect to the channel walls upon reducing the growth rate is of similar magnitude to changing confinement, but arising from a distinctly different distribution of order parameter *ϕ*. Ordering is a dynamic process that fluctuates due to coupling between density fluctuations (that allow the cells to rearrange) and strong local ordering (which transmits alignment near the walls into the bulk).

We have focused exclusively here on experimental measurements of the nematic alignment within our device. Our results provide a test for the nematodynamic equations that have successfully described ordering of bacteria in simpler systems [11]. The presence of posts within the slit for structural integrity provides a somewhat more complicated geometry than an empty slit, and the filter at one edge of our device complicates the boundary conditions when compared to an open edge or solid wall. Nevertheless, the nematodynamic equations remain two-dimensional in our system [11], suggesting that these are technical issues that can be readily resolved.

Our results, taken together with prior work [9,11,19], provide a word of caution in the use of microchemostats for single-cell biology. In some instances, for example studying cell–cell signalling, the spatial localization of the bacteria may play a role in their response [45]. In these situations, one should be careful that any ordering induced by the microfluidic system does not perturb the biology.

The general problem of nematic alignment of bacteria in confinement provides an ideal platform for studying active matter. While we have focused here on a relatively simple slit geometry, it is straightforward to fabricate more complicated topologies. For instance, two different ordered boundary layers can be frustrated in the bulk by using a trapezoidal shape instead of a square. It would also be interesting to consider the effect of undulations in the cross-sectional area. It is relatively easy to create features commensurate with the size of the domains seen in figure 5 that are not aligned with the walls. It is also possible to create features that are smaller than the typical size of the bacteria, which can strongly perturb the system [46]. Inasmuch as ordering persists far from the walls even for rather complicated chamber shapes [9], coupling geometry to the cell growth rate may lead to a very rich diversity of behaviour.

It would also be very interesting to extend the present results to investigate aspects of biofilm formation, for example by exploring a culture overexpressing adhesins. In particular, the close packing of the cells in the slit might inhibit biofilm formation by limiting diffusion of material between cells. The alignment provided by a slit microchemostat could be used to study horizontal gene transfer frequencies and see if they correlate with the ordering, and how cell metabolism, known to influence proton gradients, external pH and cell surface properties, can influence the stability of cell to cell interactions. In a similar vein, the understanding of cell arrangements is crucial for relating genetic lineages to spatial distributions, which is at the heart of various developmental biology conditions; harnessing this coupling can also enrich our potential to create functional synthetic biology systems [47]. While we have focused here on the biophysical aspects of this experimental platform, the potential for studying basic biological questions at high-throughput is a promising avenue for future research.

## Data accessibility

Cell Profiler output files, Cell Profiler pipeline and Matlab scripts for post-processing and figure production are available from the University of Minnesota Digital Conservancy through the permanent link at http://hdl.handle.net/11299/183622.
